# Dynamic pass bias control for temperature-resilient neural networks using vertical NAND flash memory

**DOI:** 10.1186/s40580-025-00513-1

**Published:** 2025-09-30

**Authors:** Sung-Ho Park, Jiseong Im, Jonghyun Ko, Joon Hwang, Yeongheon Yang, Jong-Won Back, Ryun-Han Koo, In-Seok Lee, Dongbeen Shin, Mingyun Oh, Gyuweon Jung, Jong-Ho Lee

**Affiliations:** 1 Department of Electrical and Computer Engineering and Inter-University Semiconductor Research Center (ISRC), Seoul National University, Seoul, 08826 Korea; 2 Research and Development Division, SK hynix Inc, Icheon, 17336 Korea

**Keywords:** Neural network, Neuromorphic computing, V-NAND flash memory

## Abstract

Vertical NAND (V-NAND) flash memory has emerged as a promising candidate for neuromorphic computing platforms due to its high density, scalability, and reliability. However, synaptic weights stored in V-NAND cells are highly sensitive to ambient temperature variations, resulting in significant conductance shifts that degrade the inference accuracy of neural networks. To address this challenge, we propose a dynamic pass bias (DPB) control scheme that compensates for temperature-induced weight variations without requiring memory reprogramming or additional hardware overhead. By adaptively adjusting the pass bias applied to unselected word-lines during read operations, the DPB scheme effectively stabilizes the differential conductance representation of weights under thermal fluctuations. In addition, we introduce a temperature-adaptive biasing circuit composed of a single-crystalline silicon MOSFET and V-NAND strings. Exploiting their opposing temperature-dependent resistance characteristics, this passive circuit naturally reduces the pass bias as temperature rises, enabling real-time analog compensation without explicit sensing or digital control logic. Experimental measurements on commercial V-NAND devices fabricated with over 100 WL layers reveal substantial shifts in bit-line currents with increasing temperature. Simulation results based on CIFAR-10 image classification using a VGG-11 network demonstrate that the DPB scheme significantly mitigates accuracy degradation across a wide temperature range. Notably, adjusting pass bias at lower temperatures improves classification accuracy by up to 10.5%p compared to conventional fixed-bias operations. These results highlight the effectiveness of dynamic pass bias control—both digitally and circuit-assisted—as a lightweight and scalable solution for enhancing the temperature resilience of V-NAND flash memory-based neural networks.

**Graphical abstract:**

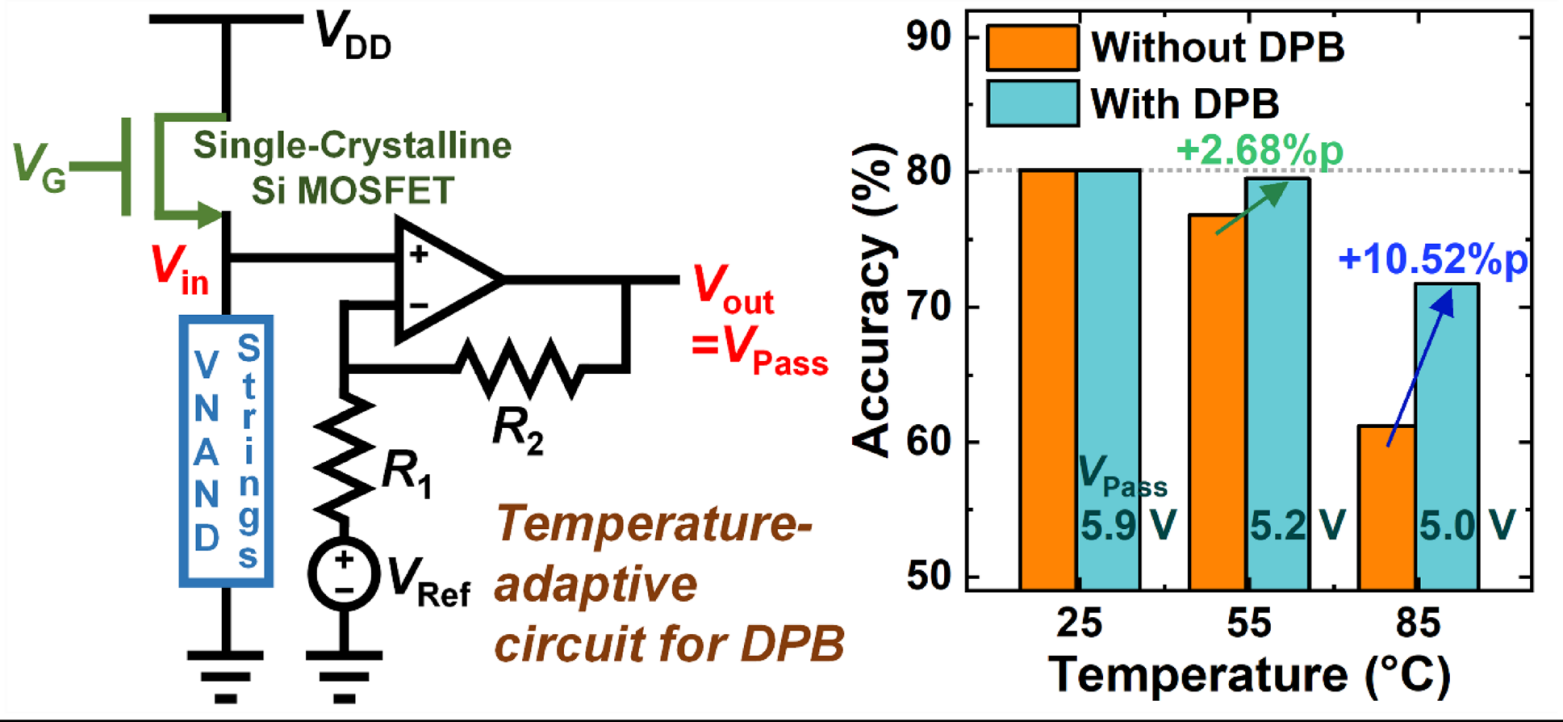

## Introduction

The proliferation of artificial intelligence and machine learning technologies has significantly increased the demand for efficient and scalable hardware platforms capable of supporting large-scale neural network computations [1, 2]. However, traditional von Neumann architectures are limited by the fundamental bottleneck of separate memory and processing units, leading to substantial energy and time penalties during data movement [3–5].

Neuromorphic computing has emerged as a promising paradigm to address this challenge by co-locating memory and computation within the same physical array [3–5]. Among various memory candidates for neuromorphic systems—including ReRAM [6], PCM [7], and FeFETs [8]—vertical NAND (V-NAND) flash memory stands out due to its high integration density, mature fabrication infrastructure, excellent retention reliability, and cost-effectiveness [9–11]. These characteristics make V-NAND flash memory an attractive candidate for realizing large-scale and energy-efficient neuromorphic hardware.

**Fig. 1:**
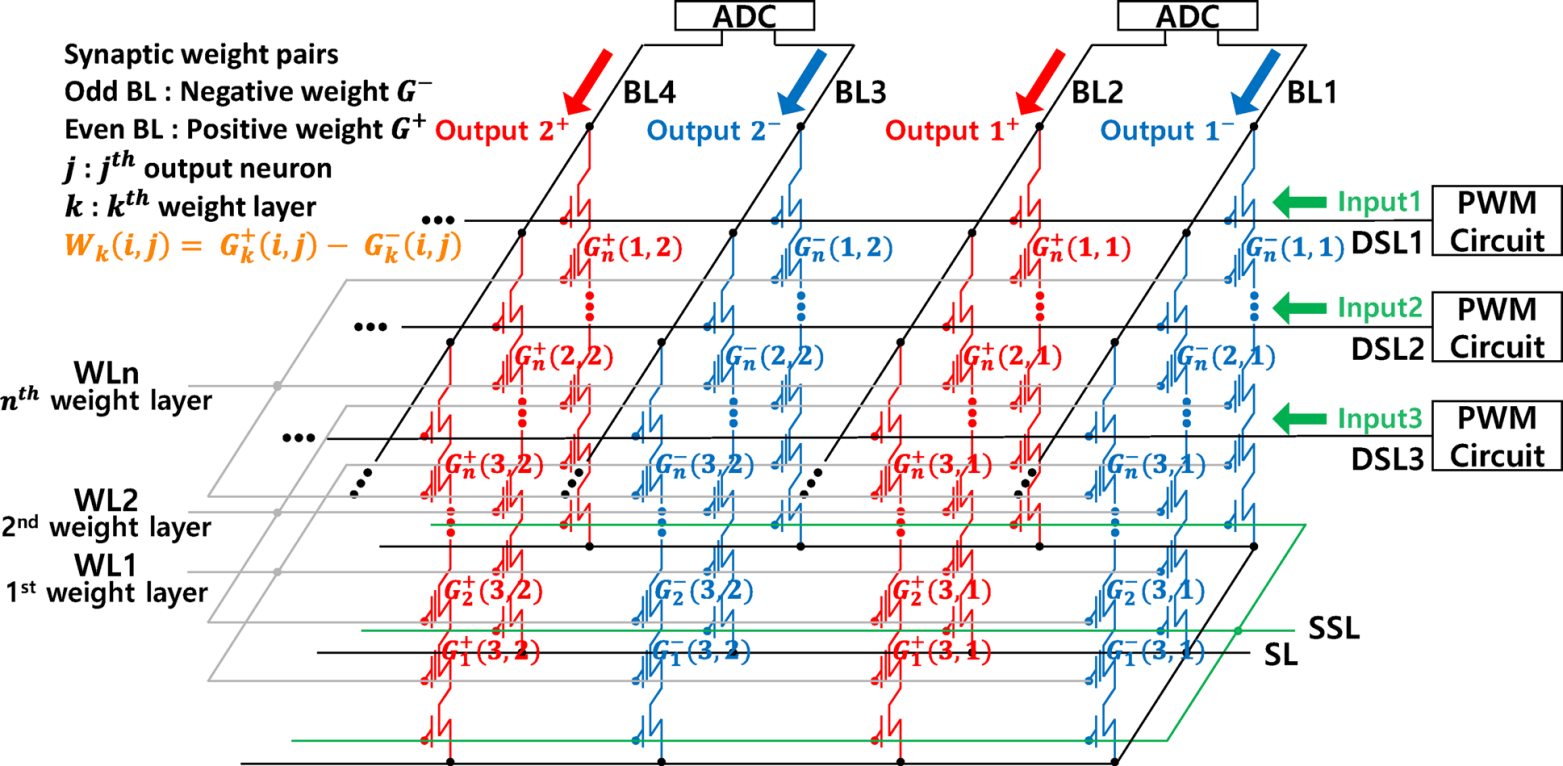
Schematic illustration of the neural network architecture implemented using V-NAND flash memory

**Fig. 2:**
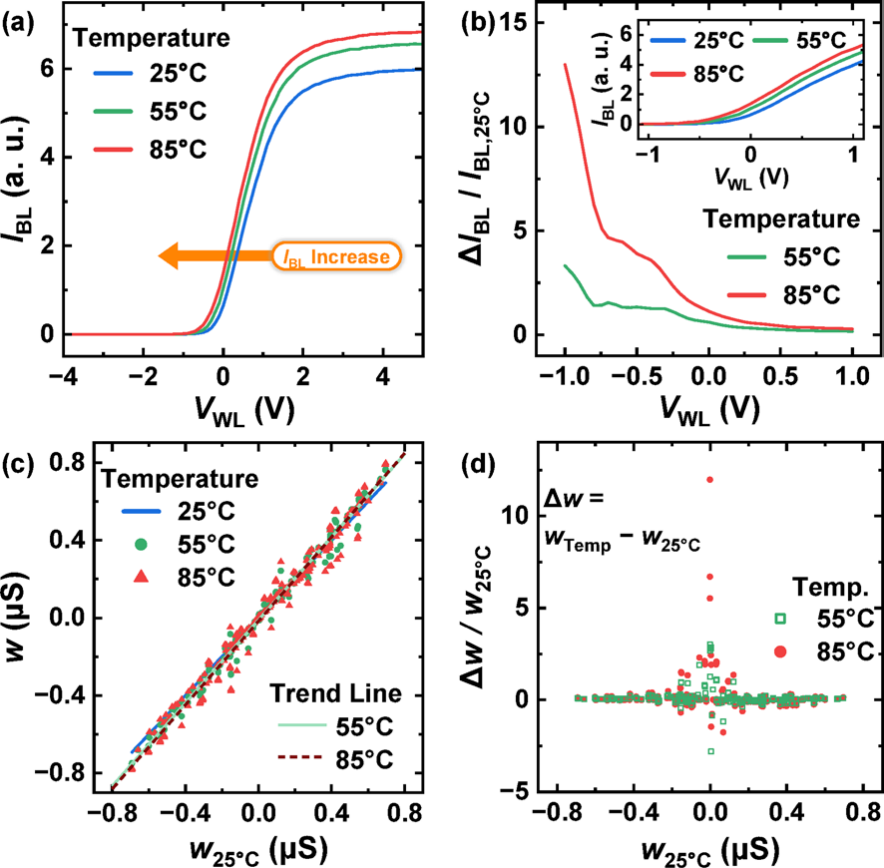
**a** Measured *I*_BL_ versus *V*_WL_ characteristics at different ambient temperatures. **b** Ratio of *I*_BL_ increase relative to *I*_BL_ at 25 °C. **c** Temperature-dependent weight values compared to those at 25 °C. A linear trend line is also shown. **d** Relative weight change as a function of initial weight

Despite these advantages, practical implementations of V-NAND flash memory-based neuromorphic systems encounter critical challenges, particularly regarding operational stability under ambient temperature variations. As temperature increases, the carrier mobility in the polycrystalline silicon (poly-Si) channel of V-NAND strings also increases, resulting in higher bit-line (BL) currents even at fixed bias conditions [12]. This temperature-induced change alters the effective conductance values of memory cells, which can distort the weight representation in neural networks and severely degrade inference accuracy.

Traditional methods to mitigate such thermal instability involve either complex circuit-based compensation mechanisms or periodic retraining of neural network weights [13]. However, as the neural network becomes more complex, the number of weights increases significantly, leading to a proportional rise in the time and energy needed for updating them. More critically, since the operating temperature of the neural network can continuously fluctuate, retraining for every temperature change is practically infeasible. As a result, these approaches often introduce significant hardware overhead, increased system complexity, or additional energy consumption—factors that counteract the primary advantages of neuromorphic computing.

In this work, we propose a dynamic pass bias (DPB) control scheme as a lightweight and effective strategy for compensating temperature-induced variations in V-NAND flash memory-based neural networks. The key idea is to dynamically adjust the pass bias (*V*_Pass_) applied to unselected word-lines (WLs) during read operations based on the detected ambient temperature. By fine-tuning *V*_Pass_, we can counterbalance the thermal effects on BL current without the need for reprogramming the memory or adding heavy compensation circuitry.

**Fig. 3:**
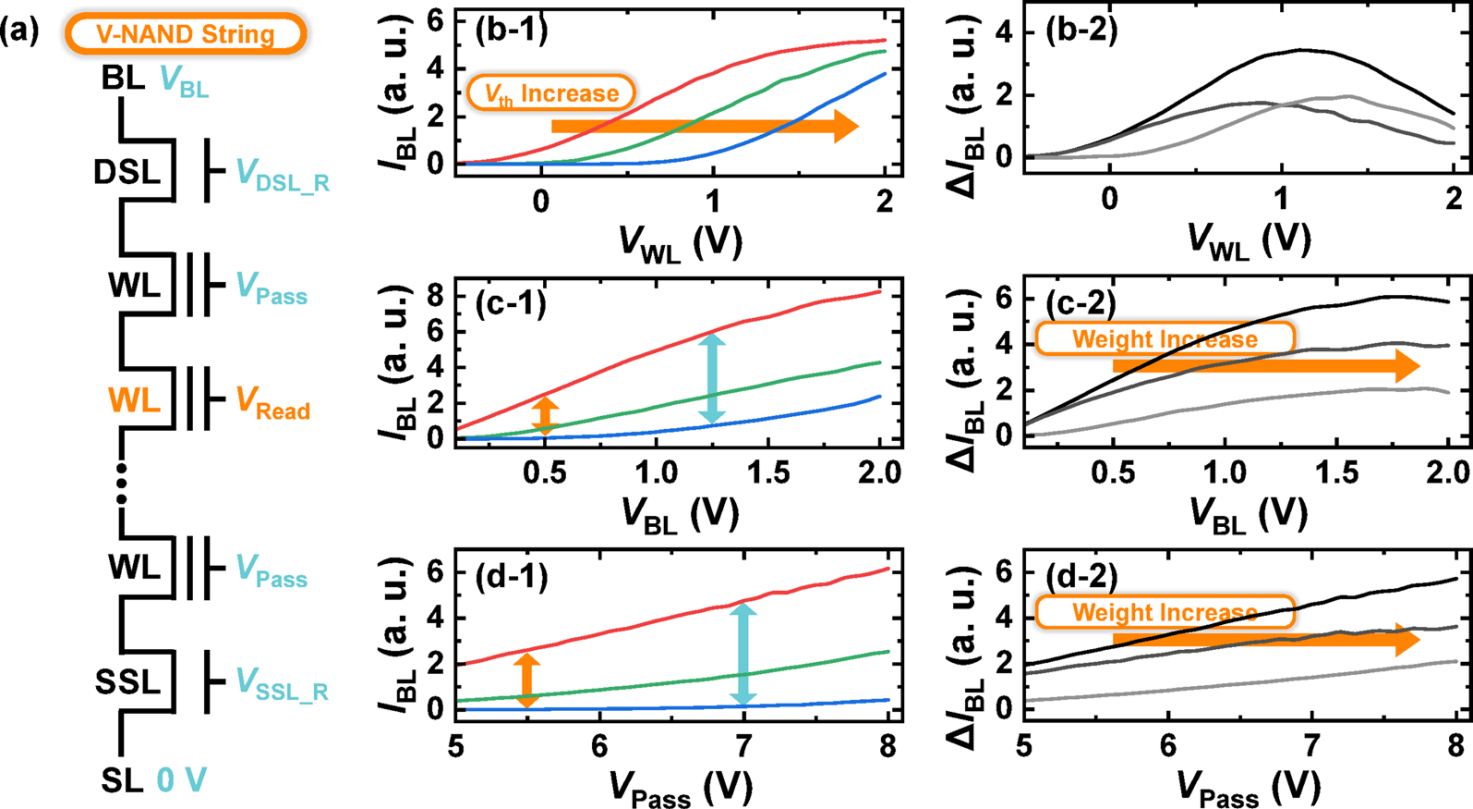
**a** Read bias configuration for V-NAND flash memory operation. **b**–**d** *I*_BL_ and BL current difference (Δ*I*_BL_) measured under varying **b** *V*_WL_, **c** *V*_BL_, and **d** *V*_Pass_ conditions. The three *I*_BL_ curves each present a different *V*_th_ state. Δ*I*_BL_ was computed by pairing, two at a time, the three *I*_BL_ curves with different *V*_th_, yielding three pairwise combinations and thus three Δ*I*_BL_ curves

To further support practical implementation, we also introduce a temperature-adaptive biasing circuit that autonomously adjusts *V*_Pass_ in real time without explicit temperature sensing or digital control. This circuit leverages the opposing temperature dependence of a single-crystalline silicon MOSFET and V-NAND strings connected in series. As a result, the voltage drop across the V-NAND string naturally decreases as temperature rises, offering an elegant analog solution for thermal compensation.

Extensive electrical measurements using commercial V-NAND flash memory devices fabricated by SK hynix, featuring over 100 stacked WL layers, reveal the impact of temperature on BL current and weight stability. Furthermore, PyTorch-based CIFAR-10 image classification simulations employing a VGG-11 architecture demonstrate that the DPB scheme significantly improves inference accuracy across a wide range of operating temperatures, highlighting its potential for enabling thermally robust and scalable neuromorphic systems.

## Results and discussion

### Neural networks using V-NAND flash memory

Figure 1 illustrates the architecture of a neural network implemented using V-NAND flash memory [14–16]. In this structure, each V-NAND string is defined by a specific combination of the drain-select-line (DSL) and the bit-line (BL), while multiple word-lines (WLs) are vertically stacked within a string, sharing a common channel [17–22]. The combination of a DSL, BL, and WL determines the basic unit of data storage, referred to as a V-NAND flash cell. The neural network based on V-NAND flash memory operates by storing weight values as conductance levels within the memory cells. To facilitate the representation of negative weights, each synaptic weight is encoded as the conductance difference between two adjacent BLs (*W* = *G*^+^−*G*^−^). Inputs to the neural network are applied to the DSLs through a pulse width modulation (PWM) circuit, which modulates the pulse width according to the input magnitude. The currents from V-NAND strings connected to the same BL are summed at the BL, enabling efficient vector-matrix multiplication (VMM) operations. The resulting BL current is subsequently processed through an analog-to-digital converter (ADC). Each WL is utilized as a distinct weight layer, and successive VMM operations are performed by sequentially reading different WLs corresponding to subsequent weight layers.

### Temperature dependence of synaptic weights

 Figure 2 depicts the temperature-dependent variations in the weights stored within V-NAND flash memory. In this work, commercial V-NAND flash memory fabricated by SK hynix, featuring over 100 WL layers, was employed for all electrical measurements. Figure 2a presents the measured BL current (*I*_BL_) versus WL voltage (*V*_WL_) characteristics at various ambient temperatures. The carrier mobility in the poly-Si channel is strongly influenced by grain boundary scattering [23]. As the temperature increases, trapped electrons at the grain boundaries gain sufficient energy to surmount or tunnel through the potential barriers, resulting in enhanced mobility and, consequently, an increase in *I*_BL_ [23]. Figure 2b shows the ratio of the temperature-induced change in *I*_BL_ relative to the reference current at 25 °C (*I*_BL,25 °C_). The increase in *I*_BL_ is more pronounced at lower *V*_WL_ values, where the device operates in the subthreshold region. These temperature-induced changes in *I*_BL_ result in unintended perturbations in the effective weights, ultimately degrading the inference accuracy of the neural network. Figure 2c displays the relationship between the synaptic weight at 25 °C and its corresponding values at elevated temperatures. As previously mentioned, in V-NAND flash memory-based neural networks, each weight is computed as the conductance difference between two adjacent BLs. The inset of Fig. 2c presents the temperature-dependent trend lines, where a steeper slope (>1) at higher temperatures reflects a larger weight error. The results reveal that larger absolute weight values exhibit greater deviation under elevated temperatures. Figure 2d quantifies the relative change in weight as a function of the baseline weight at 25 °C. Weights with small absolute values have a small denominator in the relative weight change, resulting in a large relative variation. Moreover, the deviations observed at 85 °C are significantly greater than those at 55 °C, confirming that higher ambient temperatures exacerbate weight errors in V-NAND flash memory-based neural networks. 

**Fig. 4:**
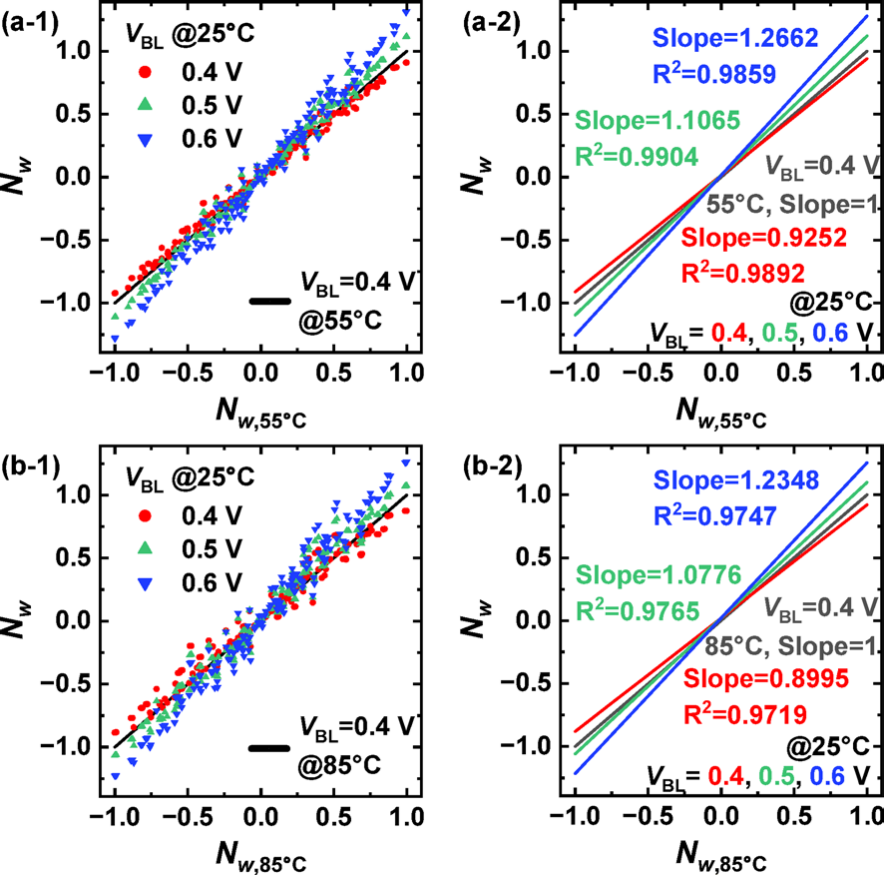
**a-1**, **a-2** Normalized weights (*N*_w_) at 55 °C compared to those at 25 °C for different *V*_BL_ values. **b-1**, **b-2** Normalized weights at 85 °C compared to 25 °C. The *V*_BL_ at high temperature was fixed at 0.4 V, and *V*_BL_ at 25 °C was varied in 0.1 V increments

**Fig. 5:**
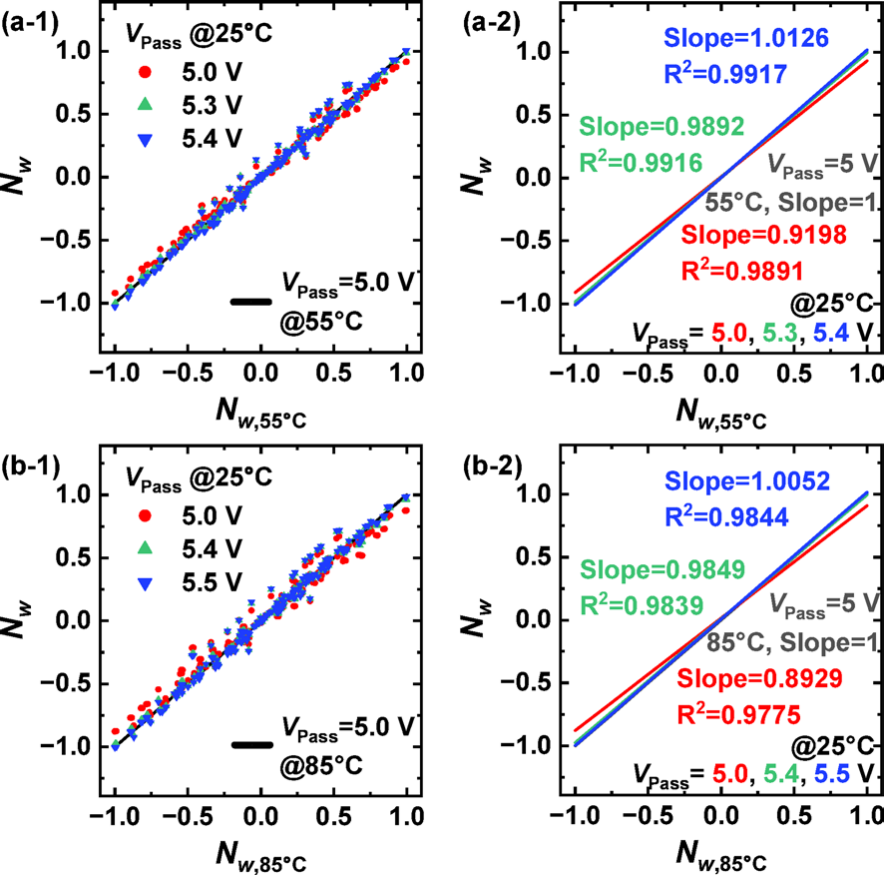
**a-1**, **a-2** Normalized weights (*N*_w_) at 55 °C compared to those at 25 °C for different *V*_Pass_ values. **b-1**, **b-2** Normalized weights at 85 °C compared to 25 °C. The *V*_Pass_ at high temperature was fixed at 5.0 V, and *V*_Pass_ at 25 °C was varied in 0.1 V increments

To compensate for temperature-induced variations in synaptic weights, the read conditions of the V-NAND flash memory can be adaptively adjusted based on ambient temperature. Figure 3a illustrates the biasing scheme applied during the read operation. A positive voltage (*V*_BL_) is applied to the BL, while the source-line (SL) is grounded to establish a current path through the selected V-NAND string. The WL corresponding to the selected V-NAND flash cell receives a designated read bias (*V*_Read_), whereas all unselected WLs are biased with a higher pass voltage (*V*_Pass_). The use of a pass bias ensures that the unselected cells remain in a conductive state, effectively transmitting the channel potential and minimizing the interference with the selected cell’s readout. Given this configuration, it is feasible to explore the temperature-dependent tuning of *V*_BL_, *V*_Read_, and *V*_Pass_ to counteract the thermally induced weight distortions. By appropriately adjusting these read voltages in accordance with ambient temperature, the deviations in current—and hence the effective conductance—can be suppressed, leading to more stable weight representation during inference.

Figure 3b–d present the *I*_BL_ and the corresponding *I*_BL_ differences (Δ*I*_BL_) as a function of *V*_WL_(= *V*_Read_), *V*_BL_, and *V*_Pass_, respectively. The read *I*_BL_s are shown for three cells with distinct conductance levels, and the current difference between any two *I*_BL_ values is denoted as Δ*I*_BL_. In V-NAND flash memory-based neural network, this Δ*I*_BL_ serves as an effective representation of synaptic weight, as it reflects the differential conductance that determines the weighted contribution during VMM. From the Δ*I*_BL_ trends under varying bias conditions, it is observed that increasing *V*_WL_ initially enhances Δ*I*_BL_ but eventually leads to a reduction, exhibiting a non-monotonic behavior. In contrast, both *V*_BL_ and *V*_Pass_ show a consistent trend where Δ*I*_BL_ increases monotonically with higher bias levels. This is because, unlike the approximately linear dependence of *I*_BL_ on *V*_BL_ and *V*_Pass_, the *I*_BL_-*V*_WL_ curve has a shape whose slope increases from zero and then decreases, causing the value to saturate. Based on this observation, it is feasible to mitigate the temperature-induced increase in effective weight magnitude by applying lower *V*_BL_ or *V*_Pass_ values during read operations at elevated temperatures.

Figure 4 shows the variation of the normalized weight (*N*_w_) as a function of *V*_BL_, along with the corresponding trend lines. The weights were normalized such that their values were distributed within the range of − 1 to 1. Figure 4a compares the *N*_w_ at 55 °C to that at 25 °C, while Fig. 4b compares the *N*_w_ at 85 °C to that at 25 °C. For the high-temperature measurements, *V*_BL_ was initially set to the typical read bias of 0.4 V, and *N*_w_ at 25 °C was measured by incrementally adjusting *V*_BL_ in 0.1 V steps. When the same *V*_BL_ as used at high temperatures was applied at 25 °C (red), a decrease in the absolute magnitude of *N*_w_ was observed, resulting in a trend line slope less than unity. Conversely, when *V*_BL_ was increased by 0.1 V to 0.5 V at 25 °C (green), the absolute value of *N*_w_ increased, yielding a trend line slope greater than unity. However, the results indicate that adjusting *V*_BL_ values in discrete 0.1 V increments is insufficient for precisely compensating for the temperature-induced weight variations.

**Fig. 6:**
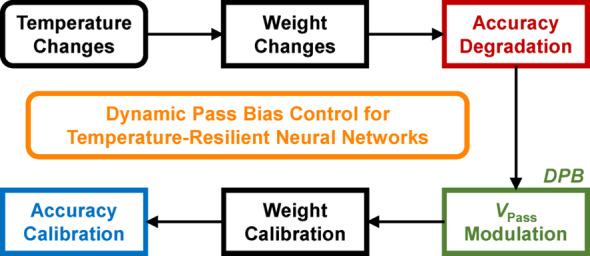
Flow diagram of constructing a temperature-resilient neural networks using the DPB scheme

**Fig. 7:**
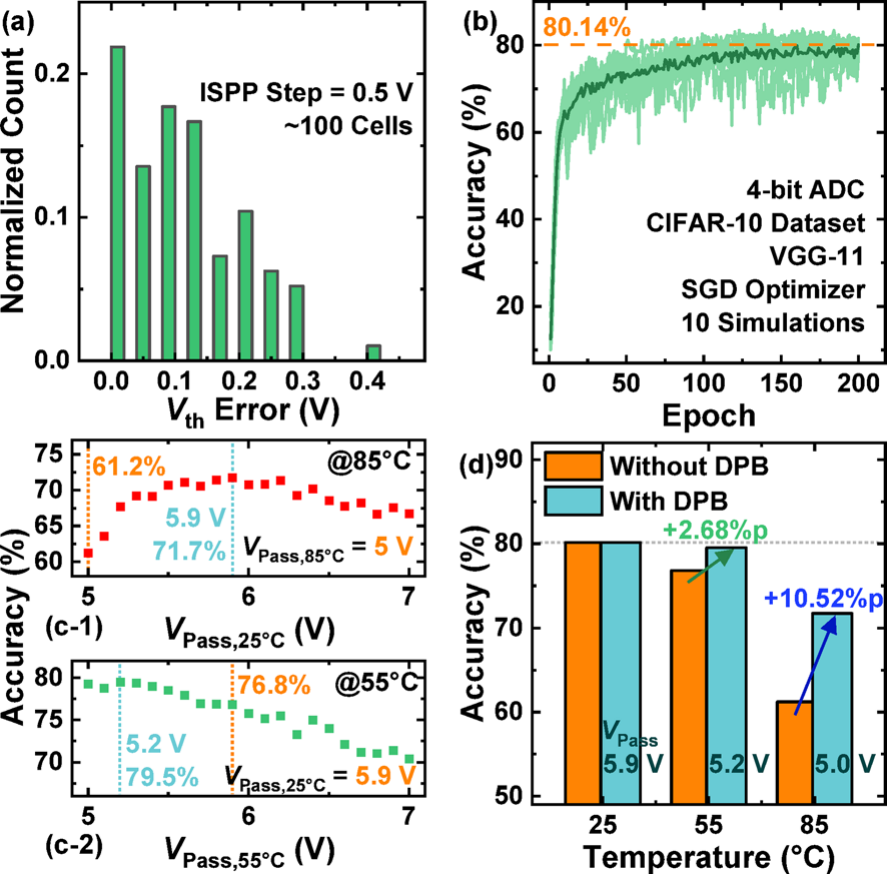
**a** Distribution of *V*_th_ tuning error after ISPP with a step voltage of 0.5 V. **b** CIFAR-10 classification accuracy over epochs using a VGG-11 network, achieving a baseline of 80.14% with 4-bit ADCs. **c-1** Accuracy at 85 °C versus *V*_Pass_ adjusted at 25 °C. **c-2** Accuracy at 55 °C with *V*_Pass_ optimization. **d** Comparison of classification accuracy across temperatures with and without DPB compensation

Figure 5 presents the variation of *N*_w_ as a function of *V*_Pass_, following a methodology similar to that of Fig. 4 but focusing on *V*_Pass_ instead of *V*_BL_. In the high-temperature conditions, *V*_Pass_ was fixed at 5 V, and at 25 °C, *V*_Pass_ was incrementally adjusted in 0.1 V steps. Unlike the case of *V*_BL_ adjustment, tuning *V*_Pass_ enabled significantly finer compensation for temperature-induced weight variations, allowing the trend line slope to be closely aligned with the ideal value of unity. These results suggest that dynamic adjustment of *V*_Pass_ is far more effective and precise than modifying *V*_BL_ for compensating weight shifts caused by temperature variations. Modifying *V*_BL_ changes the voltage difference across the both ends of the V-NAND string, which has a significant impact on the string current. In contrast, adjusting *V*_Pass_ changes the voltage applied to the WLs of unselected cells, which remain in a very low-resistance state. Since the primary factor determining the current is the *V*_Read_ applied to the WL of the selected cell—where a lower *V*_Read_ than *V*_Pass_ sets a higher resistance—the variation of *V*_Pass_ enables fine control of the current magnitude compared to *V*_BL_. Therefore, *V*_Pass_ modulation is recommended as a more reliable approach for achieving thermally robust neural network operation using V-NAND flash memory.

**Fig. 8:**
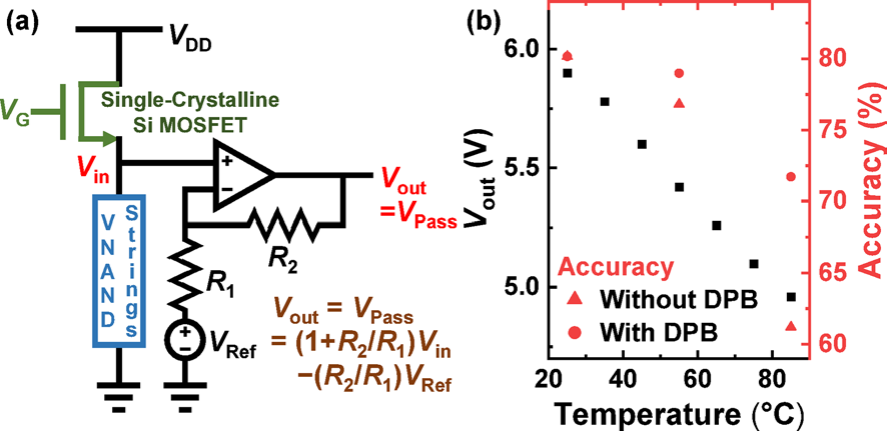
**a** Conceptual schematic of a temperature-adaptive circuit that automatically adjusts *V*_Pass_ using the opposing temperature dependence of a single-crystalline Si MOSFET and V-NAND strings. **b** Temperature-dependent output voltage (*V*_out_, corresponding to *V*_Pass_) obtained through SPICE simulation, along with the resulting CIFAR-10 classification accuracy when the temperature-adaptive circuit is applied

### Dynamic pass bias scheme

Figure 6 shows the flow diagram for constructing a temperature-resilient neural network via the DPB scheme. When temperature changes the weights and thereby reduces the neural network’s accuracy, the DPB scheme sets a *V*_Pass_ that compensates for this effect. The temperature-dependent *V*_Pass_ recalibrates the weights to values similar to the originals, thereby mitigating the accuracy degradation. Figure 7 demonstrates the effectiveness of the proposed dynamic pass bias (DPB) scheme. To evaluate the impact of DPB, CIFAR-10 image classification simulations were conducted using the PyTorch framework with a VGG-11 architecture and the stochastic gradient descent (SGD) optimizer. The ADC at the output of the neural network was configured with 4-bit resolution. Figure 7a presents the threshold voltage (*V*_th_) tuning error—defined as the deviation from the target *V*_th_—introduced by the incremental step pulse programming (ISPP) method, the conventional programming approach for V-NAND flash memory [24]. Since the weights are programmed into the V-NAND flash memory during weight transfer, the *V*_th_ error distribution was incorporated into the simulations to reflect realistic device characteristics. Figure 7b shows the CIFAR-10 image classification accuracy averaged over ten independent simulation runs as a function of the training epoch. Assuming that the weights do not change with temperature and are affected only by variations introduced during the ISPP-based transfer process, a baseline accuracy of 80.14% was achieved after 200 epochs. Figure 7c illustrates the impact of varying *V*_Pass_ levels at different temperatures on the classification accuracy. For consistency, the *V*_Pass_ at 85 °C—the highest temperature measured—was fixed at 5 V, ensuring that the *V*_Pass_ at lower temperatures remained above 5 V to maintain sufficiently low resistance in the pass cells during read operations. Figure 7c-1 shows the variation in accuracy at 85 °C when the *V*_Pass_ value used at 25 °C is altered. When *V*_Pass_ at 25 °C was increased to 5.9 V, compared to the case where both temperatures used a *V*_Pass_ of 5.0 V, an improvement of approximately 10.52% points (%p) in classification accuracy was achieved. When *V*_Pass_ =5 V at 85 °C, the normalized weight at 25 °C is best compensated for *V*_Pass_ between 5.4 and 5.5 V as shown in Fig. 5b-2, whereas the neural network accuracy is highest at 5.9 V. Although using *V*_Pass_ =5.4 ~ 5.5 V also yields substantial accuracy improvement, the reason the maximum occurs at 5.9 V appears to be that the VMM operation computed from the weight values is subsequently processed through the ADC and other blocks, leading to a slight difference from the weight-compensation result based on *V*_Pass_. Figure 7c-2 examines the accuracy at 55 °C when the *V*_Pass_ at 25 °C is fixed at 5.9 V. Adjusting *V*_Pass_ at 55 °C to 5.2 V further enhanced the accuracy by approximately 2.68%p compared to the case with *V*_Pass_ of 5.9 V at both 25 °C and 55 °C. Finally, Fig. 7d compares the CIFAR-10 classification accuracy as a function of temperature, with and without applying the DPB scheme. The absence of an accuracy difference at 25 °C between the cases with and without DPB scheme is because the reference accuracy was measured at 25 °C; thus, the weights did not change. Although the DPB-compensated accuracy does not fully recover to the 25 °C baseline of 80.14%—because the weight values corrected via *V*_Pass_ adjustment do not exactly match the pre-temperature-change weights—a substantial improvement in accuracy across different temperatures is clearly observed with the use of DPB.

### Temperature-adaptive circuit for DPB

To enable automatic compensation of the *V*_Pass_ in response to ambient temperature fluctuations, we propose a temperature-adaptive circuit structure as illustrated in Fig. 8a. The proposed circuit consists of a single-crystalline silicon (Si) channel MOSFET in series with a V-NAND flash memory block, followed by an operational amplifier (op-amp) stage that amplifies the voltage across the V-NAND strings. In this configuration, the temperature-dependent resistance behavior of each component plays a crucial role. Specifically, the resistance of the single-crystalline Si channel MOSFET increases with temperature due to enhanced phonon scattering [25], which reduces carrier mobility. In contrast, the resistance of the V-NAND string decreases with temperature, primarily because the poly-Si channel in the string exhibits increased carrier mobility at elevated temperatures. As these two opposing temperature responses are connected in series, the voltage division between them dynamically shifts. With increasing temperature, the growing resistance of the MOSFET causes a larger voltage drop across it, thereby reducing the effective voltage applied to V-NAND strings. However, the use of the MOSFET and V-NAND string alone is insufficient to produce the exact *V*_Pass_ variation required to match the compensation range observed in neural network inference. Therefore, an op-amp is introduced to amplify the temperature-dependent voltage and generate a tunable output bias. By adjusting the reference voltage (*V*_Ref_) and the resistor values (*R*_1_, *R*_2_) in the op-amp feedback network, the output voltage (*V*_Pass_) can be configured to fall within the desired range over temperature. The V-NAND string block incorporates 200 parallel-connected V-NAND strings, reflecting realistic V-NAND flash memory architecture and reducing variability among individual strings. The architecture of the proposed temperature-adaptive circuit can be used generally to generate a bias that decreases with increasing temperature; however, detailed parameters—such as resistor values, number of V-NAND strings and the dimensions of the MOSFETs—must be specified depending on the desired magnitude and range of the generated bias. The proposed temperature-adaptive circuit can be integrated into the peripheral circuitry of the V-NAND flash memory. In state-of-the-art V-NAND flash array architecture with a peripheral circuit under cell array (PUC) structure [26], including the temperature-adaptive circuit beneath the cell array together with the existing peripheral circuits can minimize the hardware area increase required to apply the DPB scheme.

To verify the feasibility of the proposed temperature-adaptive circuit for DPB, SPICE simulations were conducted using a validated V-NAND flash memory model from prior research [27]. Simulation parameters included *V*_DD_ = 10 V, *V*_G_ = 13 V, *V*_Ref_ = 6 V, and *R*_1_ = *R*_2_. The MOSFET was modeled with a channel length of 10 μm and width of 500 nm, and the WLs of each V-NAND string were biased with a pass bias of 5 V. During the operation of the proposed adaptive circuit, both the V-NAND strings and the Si MOSFET operate in saturation mode. All temperature-dependent characteristics of the circuit components were incorporated into the SPICE simulation. The simulated output voltage (*V*_out_)—the generated pass bias—exhibited a controlled decrease with temperature, as shown in Fig. 8b, aligning well with the optimal *V*_Pass_ profile required for accuracy recovery in CIFAR-10 inference (Fig. 7d). When this temperature-adaptive circuit was integrated into the DPB framework, CIFAR-10 classification simulations exhibited a substantial improvement in accuracy, confirming the practical effectiveness of the circuit-assisted compensation scheme.

This self-regulating mechanism suggests a novel approach to implementing DPB control. Rather than relying on explicit temperature sensing and digital feedback control, the circuit can inherently adjust *V*_Pass_ in real time according to the ambient thermal condition. This approach offers a compact and energy-efficient solution for enhancing the thermal robustness of V-NAND flash memory-based neural networks.

## Conclusion

In this work, we investigated the impact of temperature variations on synaptic weights stored in vertical NAND (V-NAND) flash memory for neuromorphic computing applications. Electrical measurements revealed that the bit-line current and resulting synaptic conductance exhibit significant sensitivity to ambient temperature changes, leading to substantial degradation in neural network inference accuracy. Traditional compensation techniques for thermal instability often require substantial hardware modifications or reprogramming overhead, which are undesirable for lightweight neuromorphic systems.

To address this challenge, we proposed a dynamic pass bias (DPB) control scheme that dynamically adjusts the pass bias (*V*_Pass_) during read operations based on the operating temperature. Through systematic measurements and simulations, we demonstrated that *V*_Pass_ modulation effectively counteracts temperature-induced conductance variations without modifying the stored cell states. The effectiveness of the DPB scheme was validated using CIFAR-10 image classification simulations with a VGG-11 architecture and 4-bit ADCs. The results showed that the application of DPB improved inference accuracy by up to 10.5%p compared to conventional fixed-bias operations, significantly enhancing the temperature resilience of the system.

To further support practical implementation, we also introduced a temperature-adaptive biasing circuit composed of a single-crystalline silicon MOSFET and V-NAND strings connected in series. By leveraging the opposing temperature dependencies of their resistances, the circuit inherently adjusts *V*_Pass_ without external sensing or control. This passive analog approach offers a promising direction for realizing real-time, energy-efficient DPB control without additional digital logic or feedback mechanisms.

The proposed DPB control method offers a simple, scalable, and hardware-efficient solution for maintaining stable neural network performance across a wide range of temperatures. Future work will focus on further optimizing the *V*_Pass_ adjustment algorithms, extending the approach to more complex deep learning architectures, and implementing real-time temperature-adaptive control mechanisms for fully integrated V-NAND-based neuromorphic processors.

## Data Availability

The data that support the findings of this study are available from the corresponding author upon reasonable request and with permission of SK hynix Inc.

## References

[1] Y. LeCun, Y. Bengio, G. Hinton, Deep learning. Nature. **521**, 436–444 (2015). 10.1038/nature14539 26017442 10.1038/nature14539

[2] M.I. Jordan, T.M. Mitchell, Machine learning: trends, perspectives, and prospects. Science. **349**, 255–260 (2015). 10.1126/science.aaa8415 26185243 10.1126/science.aaa8415

[3] G.W. Burr, R.M. Shelby, A. Sebastian, S. Kim, S. Kim, S. Sidler, K. Virwani, M. Ishii et al., Neuromorphic computing using non-volatile memory. Advances in Physics: X **2**, 89–124 (2017). 10.1080/23746149.2016.1259585

[4] D. Markovic, A. Mizrahi, D. Querlioz, J. Grollier, Physics for neuromorphic computing. Nat. Rev. Phys. **2**, 499–510 (2020). 10.1038/s42254-020-0208-2

[5] C.D. Schuman, S.R. Kulkarni, M. Parsa, J.P. Mitchell, P. Date, B. Kay, Opportunities for neuromorphic computing algorithms and applications. Nat. Comput. Sci. **2**, 10–19 (2022). 10.1038/s43588-021-00184-y 38177712 10.1038/s43588-021-00184-y

[6] S. Park, A. Sheri, J. Kim, J. Noh, J. Jang, M. Jeon, B. Lee, B.R. Lee et al., Neuromorphic speech systems using advanced ReRAM-based synapse, in *2013 IEEE International Electron Devices Meeting (IEDM), Washington* (2013), pp. 25.6.1–25.6.4. 10.1109/IEDM.2013.6724692

[7] O. Bichler, M. Suri, D. Querlioz, D. Vuillaume, B. Desalvo, C. Gamrat, Visual pattern extraction using energy-efficient 2-PCM synapse neuromorphic architecture. IEEE Trans. Electron. Devices **59**, 2206–2214 (2012). 10.1109/TED.2012.2197951

[8] H. Mulaosmanovic, J. Ocker, S. Muller, M. Noack, J. Muller, P. Polakowski, T. Mikolajick, S. Slesazeck, Novel ferroelectric FET based synapse for neuromorphic systems, in *2017 IEEE Symposium on VLSI Technology (VLSI), Kyoto* (2017), pp. T176–177. 10.23919/VLSIT.2017.7998165

[9] W. Jung, H. Kim, D.B. Kim, T.H. Kim, N. Lee, D. Shin, M. Kim, Y. Rho et al., A 280-layer 1 Tb 4b/cell 3D-NAND flash memory with a 28.5Gb/mm2 areal density and a 3.2GB/s high-speed IO rate, in *2024 IEEE International Solid-State Circuits Conference (ISSCC), San Francisco* (2024), pp. 236–237. 10.1109/ISSCC49657.2024.10454343

[10] S.H. Park, D. Kwon, H.N. Yoo, J.W. Back, J. Hwang, Y. Yang, J.J. Kim, J.H. Lee, Retention improvement in vertical NAND flash memory using 1-bit soft erase scheme and its effects on neural networks, in *2022 IEEE International Electron Devices Meeting (IEDM), San Francisco* (2022), pp. 5.5.1–5.5.4. 10.1109/IEDM45625.2022.10019529

[11] S.T. Lee, H. Kim, J.H. Bae, H. Yoo, N.Y. Choi, D. Kwon, S. Lim, B.G. Park et al., High-density and highly-reliable binary neural networks using NAND flash memory cells as synaptic devices, in*2019 IEEE International Electron Devices Meeting (IEDM), San Francisco* (2019), pp. 38.4.1–38.4.4. 10.1109/IEDM19573.2019.8993478

[12] S.S. Li, W.R. Thurber, The dopant density and temperature dependence of electron mobility and resistivity in n-type silicon. Solid-State Electron. **20**, 609–616 (1977). 10.1016/0038-1101(77)90100-9

[13] I. Giannopoulos, M.L. Gallo, V.P. Jonnalagadda, E. Eleftheriou, A. Sebastian, Temperature compensation schemes for in-memory computing using phase-change memory, in *2020 IEEE International Conference on Artificial Intelligence Circuits and Systems (AICAS), Genova* (2020), pp. 286–290. 10.1109/AICAS48895.2020.9074003

[14] S.H. Park, J. Ko, I.S. Lee, R.H. Koo, J.H. Kim, Y. Yang, D. Kwon, J.J. Kim et al., On-chip learning in vertical NAND flash memory using forward–forward algorithm. IEEE Trans. Electron. Devices. **71**, 3640–3644 (2024). 10.1109/TED.2024.3392170

[15] S.T. Lee, G. Yeom, H. Yoo, H.S. Kim, S. Lim, J.H. Bae, B.G. Park, J.H. Lee, Novel method enabling forward and backward propagations in NAND flash memory for on-chip learning. IEEE Trans. Electron. Devices. **68**, 3365–3370 (2021). 10.1109/TED.2021.3081610

[16] J. Ko, S.H. Park, J. Im, J. Kim, R.H. Koo, Y. Yang, J.H. Lee, Vertical NAND flash memory-based matrix-matrix multiplication scheme for energy-efficient attention score computation. Neurocomputing. **619**, 129210 (2025). 10.1016/j.neucom.2024.129210

[17] S.H. Park, H.N. Yoo, Y. Yang, J.W. Back, R.H. Koo, D. Kwon, J.J. Kim, J.H. Lee, Voltage scheme for string-select transistors to improve inhibition characteristics during 1-bit erase in vertical NAND flash. Appl. Phys. Lett. **123**, 142101 (2023). 10.1063/5.0161000

[18] C.M. Compagnoni, A. Goda, A.S. Spinelli, P. Feeley, A.L. Lacaita, A. Visconti, Reviewing the evolution of the NAND flash technology. Proc. IEEE. **105**, 1609–1633 (2017). 10.1109/JPROC.2017.2665781

[19] S.H. Park, H.N. Yoo, J.W. Back, J. Cho, Y. Yang, J. Im, R.H. Koo, J. Ko et al., Retention improvement in vertical NAND flash memory using electron back-tunneling. IEEE Electron. Device Lett. **46**, 203–206 (2025). 10.1109/LED.2024.3506507

[20] S.H. Park, H.N. Yoo, Y. Yang, J.J. Kim, J.H. Lee, Reliability improvement in vertical NAND flash cells using adaptive incremental step pulse programming (A-ISPP) and incremental step pulse erasing (ISPE). IEEE Trans. Electron. Devices. **71**, 1834–1838 (2024). 10.1109/TED.2024.3350000

[21] C. Caillat, K. Beaman, A. Bicksler, E. Camozzi, T. Ghilardi, G. Huang, H. Liu, Y. Liu et al., 3DNAND GIDL-assisted body biasing for erase enabling CMOS under array (CUA) architecture, in *2017 IEEE International Memory Workshop (IMW), Monterey* (2017), pp. 1–4. 10.1109/IMW.2017.7939067

[22] H.N. Yoo, J.W. Back, N.H. Kim, D. Kwon, B.G. Park, J.H. Lee, First demonstration of 1-bit erase in vertical NAND flash memory, in *2022 IEEE Symposium on VLSI Technology and Circuits (VLSI), Honolulu* (2022), pp. 304–305. 10.1109/VLSITechnologyandCir46769.2022.9830445

[23] J.Y.W. Seto, The electrical properties of polycrystalline silicon films. J. Appl. Phys. **46**, 5247–5254 (1975). 10.1063/1.321593

[24] K.D. Suh, B.H. Suh, Y.H. Lim, J.K. Kim, Y.J. Choi, Y.N. Koh, S.S. Lee, S.C. Kwon et al., A 3.3 V 32 Mb NAND flash memory with incremental step pulse programming scheme. IEEE J. Solid State Circuits. **30**, 1149–1156 (1995). 10.1109/4.475701

[25] G.W. Ludwig, R.L. Watters, Drift and conductivity mobility in silicon. Phys. Rev. **101**, 1699 (1956). 10.1103/PhysRev.101.1699

[26] J.W. Park, D. Kim, S. Ok, J. Park, T. Kwon, H. Lee, S. Lim, S.Y. Jung et al., A 176-stacked 512Gb 3b/cell 3D-NAND flash with 10.8Gb/mm2 density with a peripheral circuit under cell array architecture, in *2021 IEEE International Solid-State Circuits Conference (ISSCC), San Francisco* (2021), pp. 422–423. 10.1109/ISSCC42613.2021.9365809

[27] J. Hwang, H.N. Yoo, K.H. Lee, J.W. Back, M.K. Park, Y. Yang, W.Y. Choi, J.H. Lee, Accurate SPICE model for cells with tube-type poly-Si channel in cell strings of vertical NAND flash memory. IEEE Trans. Electron. Devices. **70**, 5469–5474 (2023). 10.1109/TED.2023.3308086

